# Displaced Mid-Shaft Clavicle Fractures: A Subset For Surgical Treatment

**DOI:** 10.5704/MOJ.1407.008

**Published:** 2014-07

**Authors:** P Choudhari, Chhabra

**Affiliations:** Department of Orthopaedics & Traumatology, Sri Aurobindo Institute of Medical Sciences, Indore, India; Department of Orthopaedics & Traumatology, Sri Aurobindo Institute of Medical Sciences, Indore, India

## Abstract

Recent studies have shown a high prevalence of symptomatic malunion and nonunion after nonoperative treatment of displaced midshaft clavicle fractures. We sought to study the result of surgical treatment of displaced mid-shaft fracture of clavicle. Forty mid shaft clavicle fractures in adults, displaced with initial shortening of more than 15 mm, were treated with primary internal fixation with plates. The inclusion criteria were displacement, shortening of clavicle of more than 15 mm and/or impending skin penetration. The follow-up period varied from 6 months to 24 months. Fracture union occurred in all patients (average union time 8.5 weeks). Superficial infection developed in one patient and implant was a problem in one patient. The outcome was assessed by complications, constant score and patient satisfaction questionnaire. Constant scores were excellent in the study group and 96% of the patients were satisfied with the operation. Surgical treatment in displaced fractures of the clavicle in our study produces satisfactory results.

**Key Words:**

Displaced, clavicle fracture, mid shaft and plating

## Introduction

Clavicle fractures account for 2.6% of all fractures ^1^, and 5% of adult fractures. More than 75% of clavicle fractures occur in the midshaft region. There are many treatment methods suggested for clavicle fractures although the majority are traditionally treated nonoperatively. Fractures of the middle third of the clavicle show a rotatory posterosuperior angular displacement of the medial fragment whereby the trapezuis muscle is penetrated and soft tissue interposition prevent fragments from contacting each other. In the same way overlap in comminuted fractures results in a shortening of the shoulder girdle at the fracture site, leading to poor cosmetic and functional results.

Displaced and shortened fractures of the mid-third of the clavicle are common in the young, athletic populations and are frequently high-energy injuries sustained in road traffic accidents or sports injuries. It is this subgroup of patients, namely, those with displaced and shortened midshaft fractures of the clavicle that often requires operative fixation. It introduces the key question: does acute surgical intervention result in better patient outcomes and lower or comparable complication rates as does non-operative management? Until recently, there has been no answer to this question.

In 2005, Zlowodzki et al ^2^ presented a systematic review encompassing 2144 fractures of which 97% were midshaft injuries. Their analysis of displaced midshaft fractures demonstrated a nonunion rate of 15.1% using nonoperative treatment, compared to 2.2% and 2% for plate fixation and intramedullary fixation respectively. This demonstrated a relative risk reduction of 86% to 87% for surgical intervention regarding development of nonunion. They found increasing age, fracture displacement, female gender and fracture comminution to be associated with development of non-union and long-term sequelae after non-operative treatment. Most recently, the Canadian Orthopedic Trauma Society ^3^ presented a multi-center prospective randomized controlled trial comparing initial operative plate fixation to nonoperative treatment of displaced midshaft clavicle fractures in 132 patients. Constant and DASH scores were significantly improved in the operatively treated group at all time points.

In 2006, Nowak et al 4. studied non-union and risk of sequelae in non-operatively treated clavicle fractures at nine to ten years follow up. They also defined predictable risk factors including lack of osseous contact at fracture site, a transverse fracture and increasing age that may cause complications in fracture healing and overall recovery and were considered to be indications for operative treatment.

This paper analyses the result of operative fixation of displaced clavicle fractures with an initial shortening of more than 15 mm and those with impending skin penetration. We selected these criteria as there is no previous study analysing the results of fixation based on these indications even though there is published evidence that the results of non-operative management are poor with these types of fracture.

## Materials and Methods

**Fig. 1Pre operative radiograph of left clavicle.**
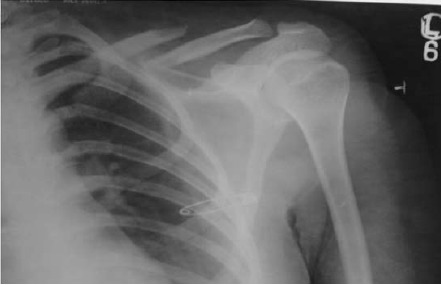


From 2007 to 2012, forty adult patients with an acute non-pathological fracture of the midshaft of the clavicle with an initial shortening of 15 mm or more or displaced fractures or fractures with impending skin penetration (Fig 1) were treated surgically at our centre.

**Fig. 2: Post- operative radiograph of left shoulder:**
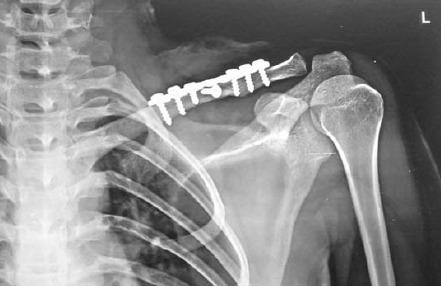


There were 29 male and 11 female patients, with an average age of 35 years. Twenty-seven were right clavicle and 13 left, fracture in dominant side in 28. Twenty-five patients had sustained the fracture following a fall, 10 from road traffic accidents and five due to assault. Thirty patients were involved in light work and 10 were heavy manual workers. All fractures were treated with open reduction and plating. The decision to operate was taken by the consultant in the trauma clinic based on the clavicle shortening of more than 15 mm or displaced fracture or impending skin penetration on initial radiographs. Patients less than 16 years of age, with open injuries, floating shoulder, and neurovascular injury were excluded from the study. The timing of the operation was three days to 1 month post- injury. The surgery was performed under general anaesthesia with patient in beach chair position. A longitudinal incision along the superior border of clavicle was made. Branches of the supraclavicular nerve were protected. The fracture was reduced and internally fixed with a reconstruction plate and screws or with locking reconstruction plate or precontoured clavicle locking plate with the aim of restoring the clavicle length and to obtain at least six cortices purchase on each side. The plate was applied on the superior surface of clavicle. (Fig 2). Lag screws were used for the large butterfly fragments. None of the cases had primary bone grafting.

Post operatively, the limb was put in a triangular sling and mobilised within pain limits straight away. The patients were encouraged to do pendulum exercises and advised to avoid lifting heavy weights at the time of discharge. The average length of inpatient stay was two days. The patients were discharged the following day and followed up at two weeks for wound healing and suture removal, and then at six, 12 and 24 weeks and as required until there was clinical and radiological union.

## Results

All the fractures healed in the anatomical position. Patient outcomes were assessed based on Constant score, complications, and patient satisfaction questionnaire. One patient had superficial infection which was controlled with antibiotics and regular dressings. Another patient underwent early plate removal due to prominence of plate. In all other patients, the plate was left in situ for a minimum of one year before removal.

The results were analysed by a physiotherapist as a neutral observer using a Biodex machine. Since it was impossible to allocate a preinjury score, the injured side was compared with the uninjured side. The Constant score ranged from 73-94 in these 36 patients and the mean score was 89.3. There were no instances of non-union, neurovascular or pulmonary complications due to surgery, or shoulder droop or loss of strength.

The patients were asked specific questions about the scar, satisfaction with the operation, level of activity and return to work, pain on sleeping on the affected side, and cosmetic abnormality.One patient considered the scar as poor. Thirty-nine patients thought that the operation helped them get back to their work and activities of daily living while one disagreed. Thirty-nine patients said that they would have the operation on the opposite side if their clavicle fractured.

## Discussion

In this retrospective assessment we present the outcome and complications of plating of displaced midshaft clavicle fractures with an initial shortening of more than 15 mm or displaced fractures or impending skin penetration. The clavicle has several important functions, each of which can be affected in non-union or malunion. The clavicle facilitates the placement of the shoulder in a more lateral position, so that the hand can be more effectively positioned to deal with the three dimensional environment. Cadaveric investigations have suggested that clavicle deformity may lead to abnormal biomechanical stresses across the shoulder girdle, including the acromioclavicular, glenohumeral and scapulothoracic joints ^5-7^. These studies provide a mechanical rationale for the idea that anatomic reduction may mitigate long-term disability. Studies of midshaft clavicle fractures with substantial shortening have reinforced these biomechanical findings by demonstrating higher patient satisfaction and improved functional outcomes after operative treatment ^3^. These studies have also shown a higher than previously recognized rate of non-union of displaced and shortened fractures treated by closed methods.

Displaced fractures of clavicle with shortening of 15 mm or more should not be treated the same way as undisplaced or minimally displaced fractures. It is very rare to achieve success with conservative treatment of such fractures. A meta-analysis of recent studies reduced the risk of non union by 86% in the operative group compared to the non-operative group ^2^.

Operative fixation allows earlier rehabilitation with a high level of patient satisfaction with respect to shoulder function. Pain relief is faster and there is no problem of having to use shoulder straps. Rigid internal fixation may also allow patients to return to certain occupations and driving vehicles earlier. Reconstruction plates can be contoured best to the three dimensional anatomy of the clavicle.

Primary fixation of the clavicle is a relatively easy procedure. Treating non-union and malunion are more difficult and challenging than fresh fractures ^8^. However, the use of open reduction in the treatment of fresh fractures remains controversial; with wide geographical and institutional variation in the choice of treatment. There is still a reluctance to treat fresh clavicle fractures with primary internal fixation in a significant number of institutions.

Stable operative fixation performed in carefully selected clavicle fractures can be a safe and effective treatment method to restore shoulder function with minimal complications ^2^. In our series the union rate was 100%. We found the superficial infection rate (2.5%) and implant problem (2.5%) similar to most of the internal fixation for trauma. Majority (96%) of the patients were satisfied with the operation. We accept that this is a relatively small series and it is a retrospective study and that all the patients were not examined prior to surgery. Having found that dealing with delayed unions and non-unions relatively difficult in the past, our department’s policy of non-surgical treatment of displaced midshaft clavicle fractures with more than 15 mm shortening was changed to open reduction and primary internal fixation with reconstruction plates in the year 2000 and the results support the change in our policy.

(Table I)

(Figure 3)

(Figure 4)

(Figure 5)

**Table IConstant score:**
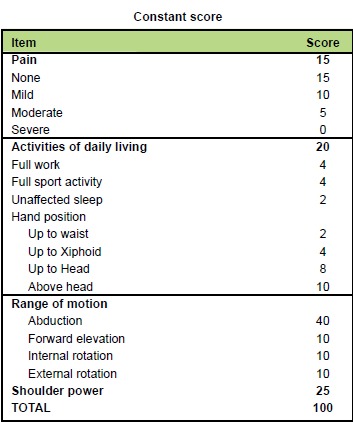


**Fig. 3: Pre and Post op radiographs of left shoulder:**
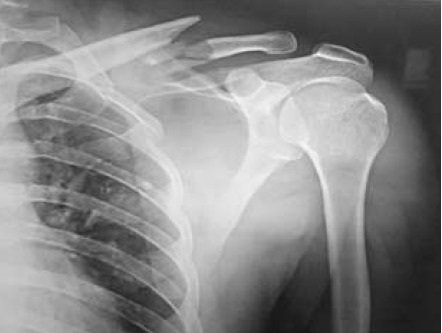


**Fig. 4: Pre and Post op radiographs of left shoulder:**
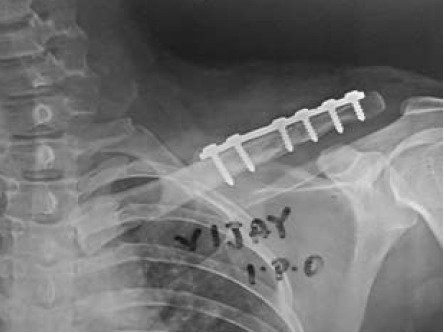


**Fig. 5: Sixth month post- operative radiograph.**
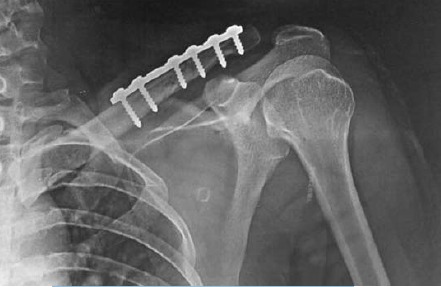


## Conclusion

The belief that clavicle fractures have a benign course and a predictably favorable outcome can be challenged. Despite the long experience with this injury, it is only recently that the sophisticated application of evidencebased techniques has called into question the traditional teaching. Most of the clavicle fracture can be treated conservatively. However, there are some fractures which require primary surgical treatment. Stable operative fixation performed in carefully selected clavicle fracture can be safe and effective treatment method to restore normal shoulder function with minimal complication.
